# Cost-effectiveness of C-reactive protein point of care testing for safely reducing antibiotic consumption for acute exacerbations of chronic obstructive pulmonary disease as part of the multicentre, parallel-arm, open, individually randomised, controlled PACE trial

**DOI:** 10.1136/bmjopen-2024-084144

**Published:** 2024-11-27

**Authors:** Bernadette Sewell, Nick Francis, Shaun Harris, David Gillespie, Janine Bates, Patrick White, Mohammed Fasihul Alam, Kerenza Hood, Christopher C Butler, Deborah Fitzsimmons

**Affiliations:** 1 Swansea Centre for Health Economics, Faculty of Medicine, Health and Life Science, Swansea University, Swansea, UK; 2 Primary Care Research Centre, School of Primary Care, Population Sciences and Medical Education, University of Southampton, Southampton, Hampshire, UK; 3 Swansea Trials Unit, Faculty of Medicine, Health and Life Science, Swansea University, Swansea, UK; 4 Centre for Trials Research, University of Cardiff, Cardiff, UK; 5 School of Life Course and Population Sciences, Kings College London, London, UK; 6 Department of Public Health, College of Health Sciences, Health Sector, Qatar University, Doha, Qatar; 7 Nuffield Department of Primary Health Care Sciences, University of Oxford, Oxford, UK

**Keywords:** health economics, patient reported outcome measures, primary health care, pulmonary disease, chronic obstructive, quality of life

## Abstract

**ABSTRACT:**

**Objectives:**

Many patients presenting with acute exacerbations of chronic obstructive pulmonary disease (AECOPD) in primary care do not benefit from antibiotics. Excessive use wastes resources, promotes antimicrobial resistance and can harm patients.

**Design:**

We conducted a within-trial economic evaluation, using a UK National Health Service perspective, as part of the multicentre, parallel-arm, open, individually randomised, controlled PACE trial.

**Setting:**

Participating general practices in primary care.

**Participants:**

PACE included 324 and 325 consenting participants presenting with AECOPD in the usual-care and CRP-guided groups, respectively.

**Intervention:**

We assessed the cost-effectiveness (CE) of a C-reactive protein point-of-care-test (CRP-POCT) in addition to usual clinical assessment to guide antibiotic prescribing for AECOPD in primary care.

**Primary and secondary outcome measures:**

A cost-effectiveness analysis (CEA) of incremental cost per 1% antibiotic consumption reduction at 4 weeks and a cost-utility analysis (CUA) at 6 months were performed, based on a modified intention-to-treat population. Sensitivity analyses assessed the impact of uncertainty on the results. CE acceptability curves represent the probability of CRP-POCT being cost-effective at different willingness-to-pay (WTP) thresholds.

**Results:**

Both groups had similar clinical outcomes, but a 20% absolute reduction in antibiotic consumption was observed in the CRP-guided group. CRP-POCT costs of £11.31 per test were largely offset by savings in healthcare resource use related to COPD. The mean incremental CE ratios of CRP-POCT were £120 per 1% absolute reduction in antibiotic consumption at 4 weeks and £1054 per quality-adjusted life-year (QALY) gained at 6 months. Sensitivity analysis showed that the CEA results were most affected by changes in healthcare costs, while CUA was sensitive due to marginal differences in costs and outcomes. There is a 73% probability of CRP-POCT being cost-effective at WTP ≤£20 000 per QALY gained.

**Conclusion:**

CRP-POCT is a cost-effective intervention for safely reducing antibiotic consumption in patients with AECOPD.

**Trial registration number:**

ISRCTN24346473

**STRENGTHS AND LIMITATIONSOF THIS STUDY**

- The first UK study that examined the cost-effectiveness of the use of C-reactive protein point-of-care test in primary care based on actual antibiotic consumption.
- Considers both a reduction in antibiotic use and no worse (non-inferior) clinical recovery.
- Robust economic evaluation, following best practices, undertaken alongside a pragmatic randomised controlled trial.
- Limited by UK National Health Service perspective, which does not consider the wider costs to the patients and society and short-term trial follow-up which does not account for the longer-term costs and outcomes associated with a chronic health condition.
- Cost-utility analysis was sensitive to changes in health-related quality of life caused by the small difference in utilities between the two groups since the PACE trial was designed to show non-inferiority in secondary outcomes rather than superiority.

## Introduction

 About 4.5% of UK adults over the age of 45 live with diagnosed chronic obstructive pulmonary disease (COPD), and about half of these experience one or more acute exacerbations of chronic obstructive pulmonary disease (AECOPD) of their disease requiring medical treatment each year.^1 2^ Of these patients, about 80% are prescribed antibiotics,^3^ with most issued in primary care.^4^ Some patients benefit from these prescriptions, but many AECOPD episodes are triggered by non-bacterial causes.^5 6^ As such, some antibiotics do not provide benefit but may damage the microbiome, drive antimicrobial resistance,^7^ risk side effects and waste scarce healthcare resources.^8^ Prescribing recommendations for primary care management of AECOPD are generally based on clinical features alone,^9^,^11^ which are subjective and provide insufficient diagnostic accuracy to predict which patients can safely be managed without antibiotics.^12^

Point-of-care tests (POCTs) for acute infections are being promoted to reduce inappropriate antibiotic prescribing, help contain antimicrobial resistance and improve patient outcomes.^13 14^ We conducted a randomised controlled trial to assess the effect of using a C-reactive protein (CRP) POCT to guide antibiotic prescribing for patients presenting with AECOPD in primary care.^15^ We demonstrated that use of this test resulted in a 20% absolute reduction in antibiotic use with no adverse effect on patient outcomes.^16^ Here, we present the findings from the health economic evaluation conducted as part of the PACE trial to assess whether CRP-POCT is a cost-effective option in addition to routine clinical assessment to guide the use and prescribing of antibiotics in patients with AECOPD in primary care.

## Methods

PACE was a multi-centre, parallel-arm, open, individually randomised (1:1) controlled trial designed to establish whether CRP-POCT in addition to usual care can safely reduce antibiotic prescribing for AECOPD while proving non-inferiority in all relevant clinical outcomes.^15 16^ The trial protocol was approved on 15 September 2014 by the Research Ethics Committee (REC) For Wales (Wales REC 6), recognised by the UK Ethics Committee Authority (REC reference: 14/WA/1106). Following informed consent, patients presenting with AECOPD to participating general practices in England and Wales were randomised to clinical management based on usual care alone (usual-care group) or usual care with the addition of a CRP-POCT (CRP-guided group). All practices were provided with a summary of national guidance on managing AECOPD.^17^

The intervention involved CRP-POCT to aid antibiotic prescribing decision at an initial consultation and in any additional consultations for AECOPD within 4 weeks post-randomisation using standard guidance for interpretation of results.^15^ The usual-care group had no CRP-POCT during the 4 week post-randomisation period.

The co-primary outcomes comprised patient-reported antibiotic consumption for AECOPD within 4 weeks post-randomisation and COPD health status assessed by the Clinical COPD Questionnaire (CCQ) at 2 weeks post-randomisation. Analysis was based on a modified intention to treat (MITT) population (ie, all randomised participants who provided outcome data) with 324 participants in the usual-care group and 325 participants in the CRP-guided group. Patients and public contributors were involved in the design, conduct, reporting and dissemination plans for this research. Further details of the PACE trial have been previously reported.^15 16^

We conducted a within-trial economic evaluation from a UK National Health Service (NHS) perspective as per the health economic analysis plan (available from the authors by request). We assessed CRP-POCT implementation costs in primary care and subsequent healthcare costs, related to COPD and respiratory conditions, within 6 months post-randomisation. A cost-effectiveness analysis (CEA) was conducted based on the co-primary outcome of antibiotic consumption at 4 weeks. A cost-utility analysis (CUA) was performed at 6 months calculating the incremental cost per quality-adjusted life-year (QALY) gained. A range of sensitivity analyses assessed the impact of changing prespecified parameters on the base-case economic evaluation. As the time horizon was less than 12 months, no discounting was required. Excel 2010, SPSS 25 and STATA V.14.3 were used for analyses.

### C-reactive protein point-of-care test (CRP-POCT) implementation and chronic obstructive pulmonary disease (COPD)-related healthcare costs

We included costs of the CRP-POCT implementation in primary care, costs of medications prescribed (including antibiotics, oral corticosteroids and inhaled medications) and costs of primary and secondary healthcare resources related to COPD. Costs were expressed as 2015/2016 UK Pound Sterling (£), inflated and converted appropriately where required.^18^ Resource use resulting from CRP-POCT implementation (including materials, consumables, staff time and training) was estimated through interviews and direct communications with participating general practice staff, the CRP-POCT manufacturer and the trial team, and using data collected during the trial (eg, frequency of repeat testing). We obtained unit costs of materials and consumables directly from the manufacturer and online wholesale catalogues. Staff costs were estimated using published unit cost.^19^ New prescriptions of antibiotics, oral steroids and inhaled medication for treatment of COPD were recorded routinely during the 6-month note review within the trial. Unit costs were obtained from the Monthly Index of Medical Specialities^20^ and the British National Formulary.^21^ We costed individual prescriptions on dose, duration and daily frequency. Where information was missing or could not be extrapolated, the most commonly prescribed antibiotic (amoxicillin 500 mg, 21 tablets), oral steroid (prednisolone 5 mg, 56 tablets) and inhaled medication (salbutamol metered dose inhaler 100 mcg) were assumed. For inhaled medications, we assumed that a new prescription was issued where medication was increased at the index consultation. We calculated prescription costs over the 4-week and 6-month post-randomisation periods assuming that 1/6 of all medication prescriptions recorded in the 6-month review period would have occurred in the first 4 weeks. This assumption was tested in sensitivity analyses.

Healthcare resource use data (including primary care consultations, A&E visits, outpatient appointments and in-patient stays) were collected using an adapted Client Service Receipt Inventory (CSRI) integrated in the 4-week case report form (CRF) and a 6-month note review. We assessed the differences in healthcare use profile due to COPD and other respiratory conditions between the CRP-guided and usual-care groups. Where the CSRI had one or more items completed (ie, value of ‘0’ or greater), it was assumed to be completed and blank items imputed with zero. Where the CSRI was marked as not done or fully incomplete, data were assumed missing and addressed appropriately. Costs were assigned using published unit costs.^19 22^ Outpatient visits and inpatient stays were costed individually according to the reasons for healthcare contact, length of stay and specialty/department visited as recorded in the trial CRFs. The mean healthcare costs in both groups were summated based on all available cases, and non-parametric Mann-Whitney U tests were used to compare between-group differences. Non-parametric bootstrapping was employed to derive 95% CIs to account for the skewness of cost data.

### Outcomes

The co-primary clinical outcomes used in the trial were patient-reported antibiotic consumption for AECOPD within 4 weeks post-randomisation and COPD health status measured by the CCQ at 2 weeks post-randomisation. For the cost-effectiveness analysis, we used patient-reported antibiotic consumption to produce an absolute percentage reduction in antibiotic consumption at 4 weeks.

Participants’ health-related quality of life (HRQoL) was assessed using the European Quality of Life 5 Dimensions 3 Level Version (EQ-5D-3L) during the internal pilot (n=60) and the European Quality of Life 5 Dimensions 5 Level Version (EQ-5D-5L) questionnaire for all trial participants beyond the pilot at baseline and 6 months post-randomisation. The descriptive system was used with the UK social tariff for the EQ-5D-5L and EQ-5D-3L, respectively, to generate a utility score for each trial participant at each time point. In accordance with the National Institute for Health and Care Excellence (NICE) recommendations at the time of analysis,^23^ EQ-5D-5L data were mapped to the EQ-5D-3L valuation set using the crosswalk index value calculator, which allowed for a practical approach to handling the different versions of the EQ-5D between internal pilot and full trial. QALYs were calculated based on the utility scores at baseline and 6 months using the area-under-the-curve approach and linear interpolation.^24^ We used the EQ-5D MITT population, which included all patients who had a complete baseline questionnaire and one or more complete follow-up questionnaires, using a logistic-regression model, adjusted for the number of Anthonisen criteria before randomisation, with the potential correlated nature of the data from the patients within practices taken into account.

### Missing data

While the descriptive analysis of health and care resources was undertaken based on available cases, the comparative analysis to establish differences between groups used the MITT population. Assuming data were missing at random, multiple imputation was performed to account for missing data using chained equations. Predictive mean matching (PMM) was used for cost, medication days and utility variables; logistic regression was deemed appropriate for antibiotic prescription variables. PMM for continuous variables was used to avoid the imputation of values outside of plausible ranges (eg, utility values greater than one and costs less than zero). A total of 20 imputations were added, and results were combined using Rubin rules.^25^ The imputation model used site, allocation and baseline cost and utility variables as covariates.

### Cost-effectiveness analyses

The CEA expressed the incremental cost required to achieve a 1% absolute reduction in the number of people consuming at least one dose of antibiotics in the 4 weeks following randomisation. An incremental cost-effectiveness ratio (ICER) was estimated as the difference between groups in mean total costs divided by the difference in mean reduction in antibiotic consumption between groups. No established willingness-to-pay (WTP) threshold values for the cost-effectiveness (CE) in reduction of antibiotic consumption exist.

For the CUA, we used threshold values recommended by NICE. Generally, where an intervention is less costly but more clinically effective compared with all other relevant alternatives, the intervention dominates the alternatives. Where an intervention is more expensive and less clinically effective, the intervention is dominated. Where the intervention has an ICER of £20 000 or less per QALY gained compared with the next best alternative, it may be considered cost-effective (ie, worth the extra cost of producing one extra QALY or the extra savings achieved by sacrificing one additional QALY). No conditions for non-inferiority were applied.

### Sensitivity analyses

Deterministic univariate sensitivity analyses changed test, medication and healthcare costs, and outcomes individually within plausible ranges (eg, 95% CIs, ±30%). To address joint uncertainty in costs and effects, probabilistic sensitivity analysis, using non-parametric bootstrapping, assessed the impact on the ICER from 1000 random resamples with replacement with results presented on cost-effectiveness (CE) planes. CE acceptability curves described the probability of CRP-POCT being cost-effective, compared with usual care, at different WTP thresholds. No subgroup analyses were performed.

## Results

### C-reactive protein point-of-care-test (CRP-POCT) implementation costs

The total cost of CRP-POCT was estimated to be £11.31 per test (see online supplemental table A). Every CRP-guided group patient received one test at the index consultation. In the 4 weeks after randomisation, a total of 20 CRP-POCT tests were conducted on 18 patients in the CRP-guided group. This increased the per-patient cost of CRP testing at the 4-week follow-up point (including baseline) to £12.08 (SD=£3.23).

### Cost of chronic obstructive pulmonary disease (COPD)-related medication

A breakdown of the mean costs for all COPD-related prescribed medications can be found in table 1.

**Table 1:** Cost of acute exacerbations of chronic obstructive pulmonary disease–related medications, primary and secondary care for PACE trial participants

	CRP-POCT group (n=325)	Control group (n=324)	Difference (95% CI)*	p value
**Antibiotics**				
Mean antibiotic cost at index consultation (£), per patient (SD)	0.63 (0.69)	0.91 (0.72)	−0.28 (−0.38 to −0.16)	>0.001
Mean antibiotic cost at 6-month review (£), per patient (SD)	2.20 (4.69)	2.05 (2.78)	0.15 (−0.41 to 0.79)	0.194
**Oral steroids**				
Mean oral steroid cost at index consultation (£), per patient (SD)	0.75 (0.77)	0.74 (0.73)	0.02 (−0.06 to 0.17)	0.949
Mean oral steroid cost at 6-month review (£), per patient (SD)	1.10 (1.92)	1.26 (2.88)	−0.16 (−0.54 to 0.28)	0.292
**Inhaled medications**				
Mean inhaled medications cost at index consultation (£), per patient (SD)	3.14 (8.83)	3.10 (8.41)	0.05 (−1.43 to 1.46)	0.875
Mean inhaled medications cost at 6-month review (£), per patient (SD)	10.05 (18.65)	7.74 (16.20)	2.30 (−0.23 to 5.28)	0.134
**Total medication cost**				
Mean total medication cost at index consultation (£), per patient (SD)	4.51 (8.76)	4.70 (8.39)	−0.21 (−1.68 to 1.25)	0.116
Mean total medication cost at 6-month review (£), per patient (SD)	13.35 (19.55)	11.05 (17.00)	2.30 (−0.61 to 5.21)	0.371
**Primary care costs at 6-month review (£), per patient (SD**)				
GP visits at surgery	45.95 (55.06)	50.07 (59.78)	−4.12 (−13.10 to 4.70)	0.818
Nurse visits at surgery	7.31 (10.44)	6.67 (11.83)	0.64 (−1.26 to 2.48)	0.086
GP visits at home	3.08 (18.66)	4.50 (22.53)	−1.43 (−4.76 to 2.02)	0.505
Nurse visits at home	0.00 (0.00)	1.10 (13.12)	−1.10 (−2.83 to −0.08)	0.131
GP phone consultations	10.89 (25.96)	11.23 (28.26)	−0.34 (−4.85 to 4.01)	0.670
Nurse phone consultations	0.82 (2.63)	0.76 (2.79)	0.06 (−0.38 to 0.48)	0.499
Other contacts	0.09 (0.94)	0.16 (1.63)	−0.06 (−0.30 to 1.33)	0.688
Total cost of primary care use per patient	68.13 (72.34)	74.49 (85.37)	−6.35 (−18.91 to 6.11)	0.627
**Secondary care costs at 6-month review (£), per patient (SD**)				
Accident and emergency visits	16.26 (61.07)	14.60 (55.35)	1.66 (−7.38 to 11.01)	0.971
Outpatient visits	24.06 (77.74)	36.84 (105.53)	−12.79 (−28.04 to 1.91)	0.153
Inpatient stays	134.16 (855.00)	123.57 (625.23)	10.59 (−103.27 to 133.12)	0.691
Total cost of secondary care use per patient	174.48 (911.54)	175.01 (669.57)	−0.53 (−123.23 to 130.94)	0.333
**Total healthcare costs in the 6-month review period (£), per patient (SD**)				
Total cost (based on all available cases) and including intervention cost	294.14 (906.15)	287.33 (673.70)	6.81 (−116.49 to 130.11)	0.505

* 95% CIs are based on non-parametric bias-corrected accelerated 5000 bootstrapped resamples. P values are based on non-parametric Mann-Whitney U tests comparing median differences between groups.

CRP-POCTC-reactive protein point-of-care-testGPgeneral practitioner

Considering all available cases (n=649), 47.7% of patients in the CRP-guided group were prescribed antibiotics at their baseline GP consultation following CRP testing, compared with 69.4% in the usual-care group. The most commonly prescribed antibiotics were amoxicillin (59.5%), doxycycline (24.0%) and clarithromycin (12.8%). In the 6 months following the baseline GP consultation (n=606), 12.8% fewer patients received antibiotic prescriptions in the CRP-guided group compared with the usual-care group. However, patients in the CRP-guided group were issued on average 0.21 more prescriptions than those in the usual-care group (2.33 vs 2.12) and received more expensive antibiotic formulations (mean £1.66 per prescription compared with £1.53 in the usual-care group), resulting in a £0.15 increase in cost of antibiotics per patient in the CRP-guided group (p=0.194). Overall, when initial consultation and 6-month review period prescriptions were combined, there was a statistically significant, but small reduction in the mean cost of antibiotics in the CRP-guided group by £0.13 (95% CI −£0.72 to £0.46) per patient (p=0.031).

Oral corticosteroids were prescribed to 54.9% of patients in the CRP-guided group and 55.6% in the usual-care group at baseline, with marginally more prescriptions per patient in the CRP-guided group. The difference in cost of oral steroids during the 6-month review period was not statistically significant (table 1).

In the CRP-guided group, 21.9% of patients were prescribed new inhaled medications or had their existing prescription increased at baseline compared with 22.7% in the usual-care group, with more prescriptions per patient (1.23 vs 1.18). During the 6-month follow-up period, 5.4% more inhaled medication was prescribed to patients in the CRP-guided group than in the usual-care group. Combining baseline and 6-month review period prescriptions, there was no significant difference in total inhaler cost (mean £2.21; 95% CI −£0.75 to £5.18; p=0.375) per patient between the groups.

### Cost of chronic obstructive pulmonary disease (COPD)-related healthcare use

Fewer patients had COPD-related general practitioner (GP) home visits, nurse home visits and GP phone consultations in the CRP-guided group compared with control (see table 1). GP surgery consultations for COPD were 2.7% less frequent (58.6% vs 61.3%), with slightly fewer visits per patient (2.18 in CRP-guided group compared with 2.27 in usual-care group) resulting in an overall non-significant difference in primary care cost due to COPD in the CRP-guided group of −£6.35 (95% CI −£18.91 to £6.11, p=0.627) per patient. Mean hospital inpatient stay duration was 1.68 days longer per admitted patient (95% CI −1.92 to 5.28, p=0.617) in the CRP-guided group (6.42 days vs 4.74 days), but the cost difference was not statistically significant. Total COPD-related secondary care cost was marginally lower in the CRP-guided group because of a lower number of outpatient appointments (see table 1).

**Figure 1:**
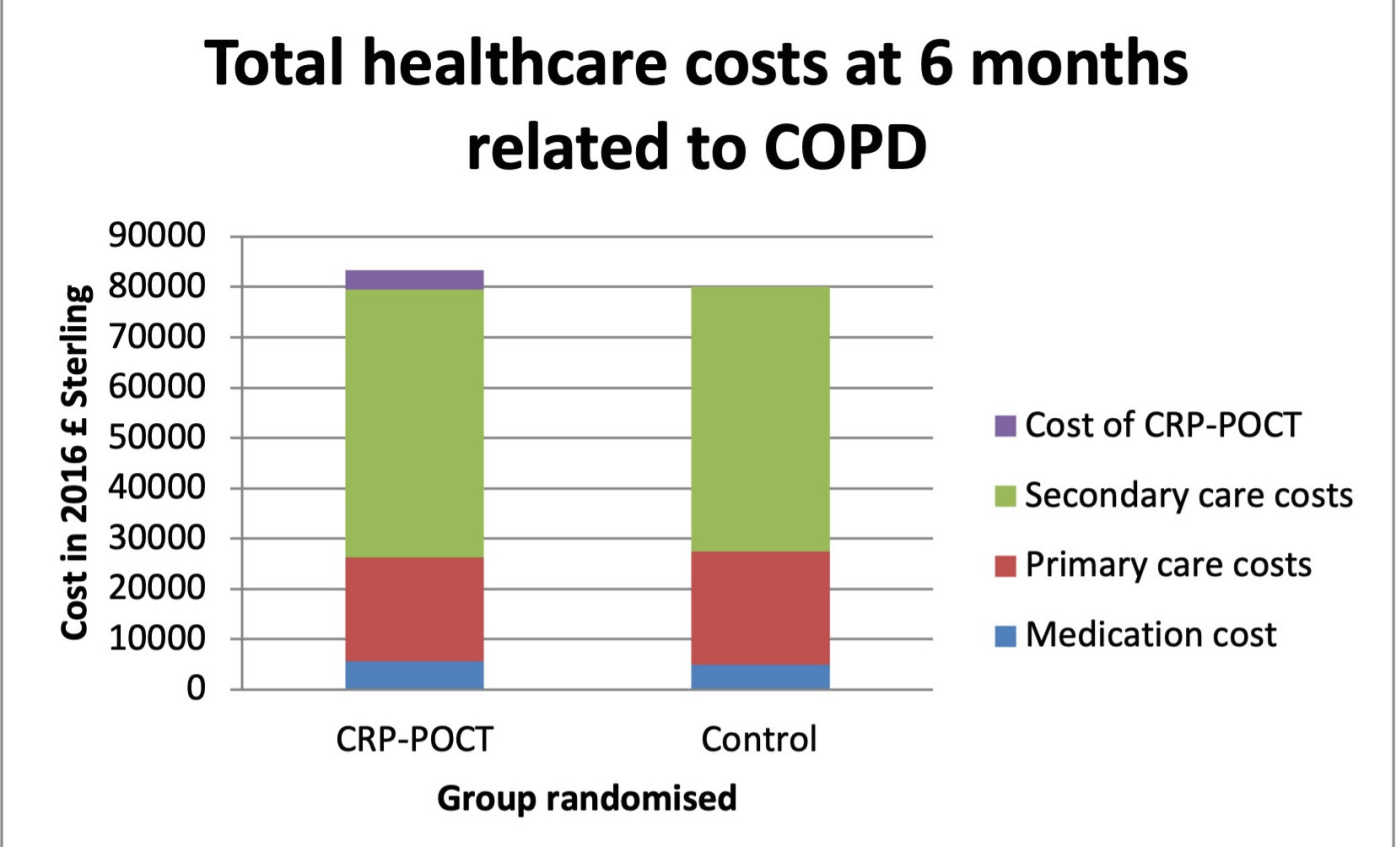
Total cost associated with chronic obstructive pulmonary disease (COPD) and other respiratory conditions (including baseline costs) during 6-month study period for C-reactive protein point-of-care-test (CRP-POCT) and control group.

Total COPD-related healthcare cost (including the index consultation and 6-month follow-up period) was £294.14 (SD=£906.15) per patient in the CRP-guided group and £287.33 (SD=£673.70) per patient in the usual-care group, with no evidence of statistical difference (95% CI −£116.49 to £130.11, p=0.505; see figure 1).

### Clinical outcomes

Of the 649 participants randomised, 537 contributed to the primary analysis of antibiotic consumption (82.7%). The odds of consuming an antibiotic for AECOPD during the first 4 weeks following randomisation were 69% lower in participants allocated to the CRP-guided group compared with usual care (adjusted OR=0.31; 95% CI 0.20 to 0.47; p<0.001). In the usual-care group (n=274), 77.4% of patients consumed antibiotics compared with 57.0% in the CRP-guided group (n=263).^16^

Health utility was non-inferior in the CRP-guided group compared with usual care when averaged across follow-up time points (adjusted mean difference=0.03, 95% CI −0.04 to 0.09, p=0.384). The mean number of QALYs gained over the 6-month review period was 0.2915 (SD=0.1240) in the usual-care group and 0.3000 (SD=0.1275) in the CRP-guided group.

### Cost-effectiveness analysis

The mean cost for the MITT population (n=537) at the 4-week follow-up point (including baseline) was £94.40 (SD=£142.39) in the CRP-guided group (n=274) and £70.06 (SD=£83.44) in the usual-care group (n=263). This represents an incremental cost of £24.34 (95% CI £4.65 to £44.03, p=0.015) per participant in the CRP-guided group. Considering a reduction of antibiotic consumption of 20.34% in the CRP-guided group, the mean ICER is £120 per 1% absolute reduction in antibiotic consumption.

**Figure 2:**
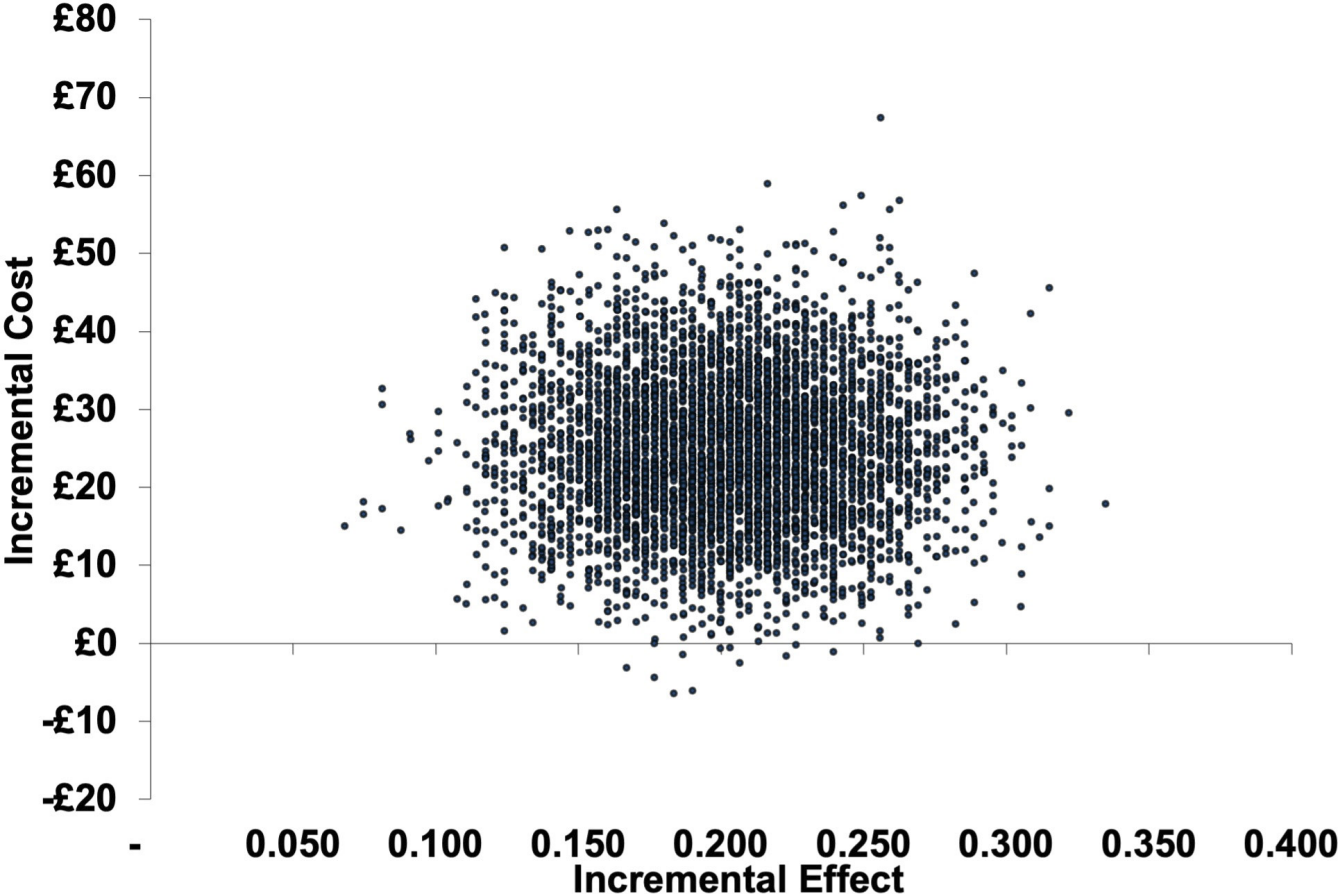
Cost-effectiveness plane depicting incremental cost per 1% absolute reduction in antibiotic consumption calculated in 1000 bootstrapping iterations.

In all sensitivity analyses, ICERs ranged between £114 and £152 per 1% reduction in antibiotic consumption. ICERs were generally robust but most affected by changes in healthcare costs and antibiotic consumption. Probabilistic sensitivity analysis following bootstrapping showed that the majority of plausible ICERs indicated the intervention being more costly and more effective (see figure 2) with a probabilistic mean ICER of £125 (95% CI −£42.00 to £518.14).

### Cost-utility analysis

There was no evidence of inferiority in cost outcomes in the CRP-guided group, with total COPD-related cost at 6 months (including baseline) for the EQ-5D MITT population of £309.93 (SD=£941.03) per person in the CRP-guided group (n=301) and £300.97 (SD=£697.08) in the usual-care group (n=301). Furthermore, no evidence of a difference in QALYs was found. However, despite the initial premise of non-inferiority, a marginal QALY increase of 0.0085 (95% CI −0.0117 to 0.0286, p=0.760) in the CRP-guided group and slightly higher costs resulted in a base case ICER of £1054 per QALY gained.

Results remained reasonably robust during deterministic sensitivity analysis when subjected to changes in the cost and QALY inputs with ICERs between £847 and £1323 but most sensitive to healthcare cost and QALY gain due to the small between-group differences.

**Figure 3:**
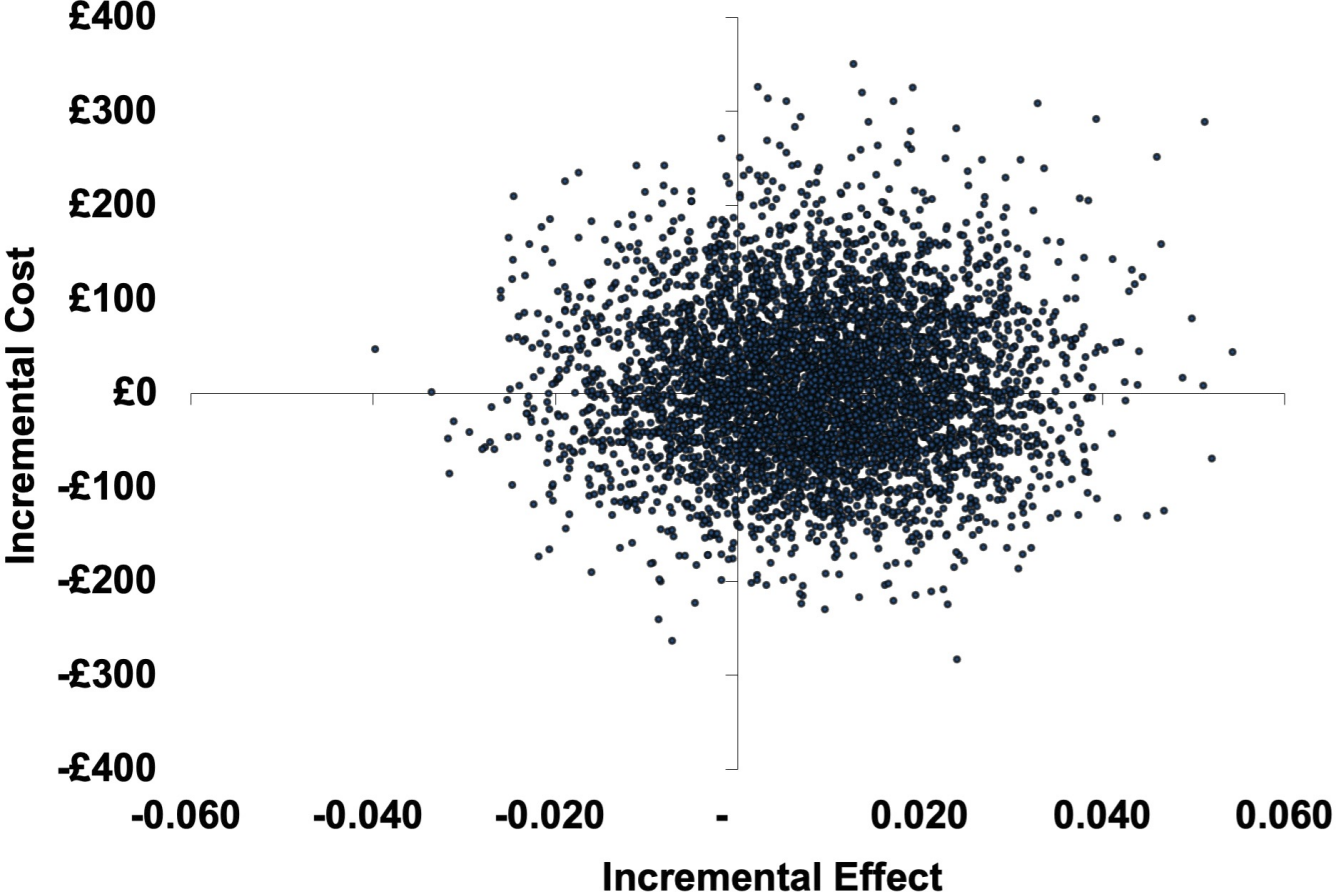
Cost-effectiveness plane (modified intention to treat analysis) for the base case cost-utility analysis (incremental cost per quality-adjusted life-year gained).

**Figure 4:**
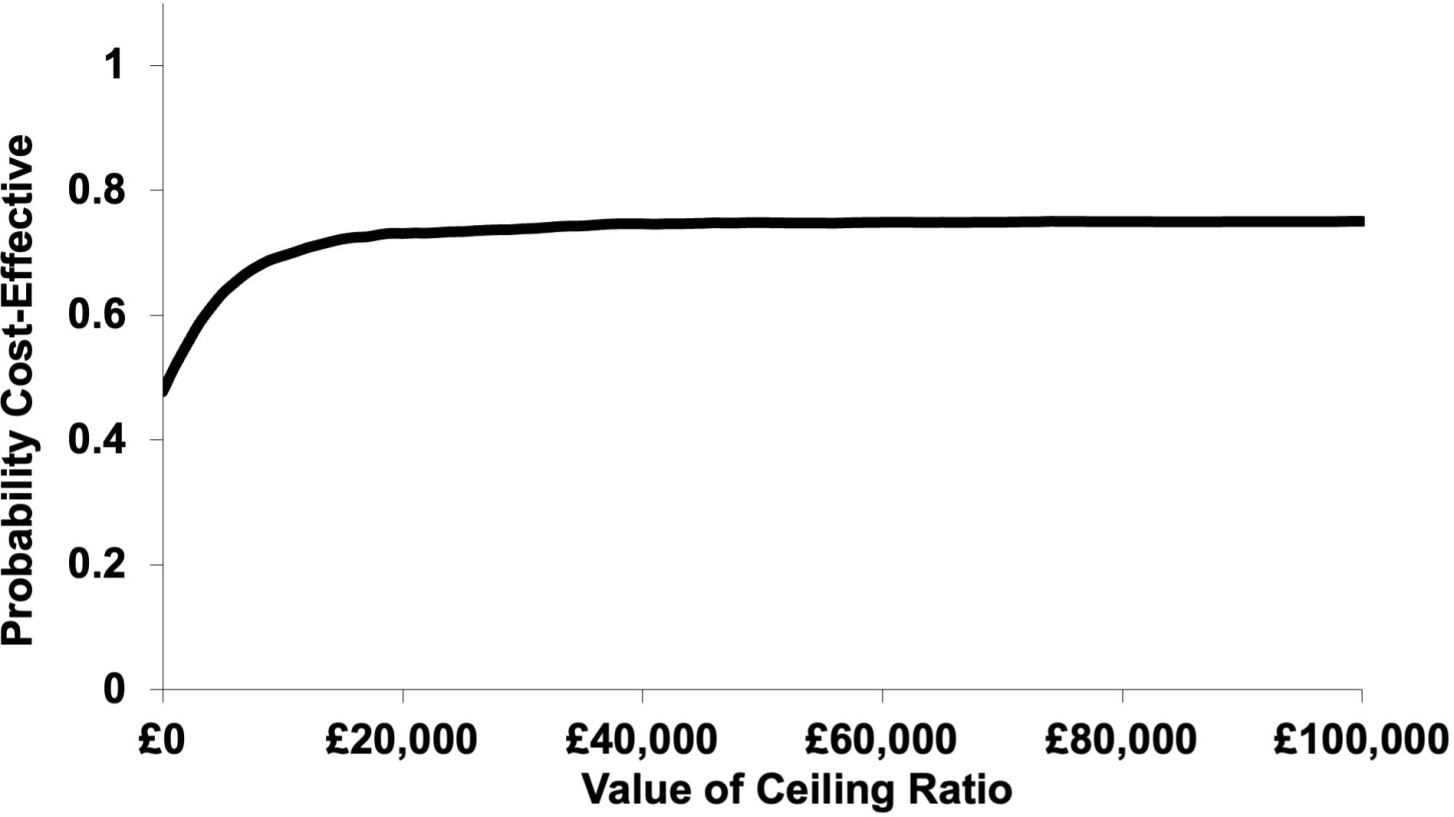
Cost-effectiveness acceptability curve (modified intention to treat analysis) for the base case cost-utility analysis (incremental cost per quality-adjusted life-year gained).

In the probabilistic sensitivity analysis, most results found CRP-POCT to be more costly but also more effective. However, results are distributed across all quadrants of the CE plane resulting from the small differences in costs and QALYs between the two groups (see figure 3). Overall, the mean probabilistic ICER was £1489 (95% CI −£61 895 to £65 848) per QALY gained with a probability of CRP testing to be cost-effective at a WTP threshold of £20 000 per QALY gained of 73.1% (see figure 4).

Repeating the CUA using the ITT population after multiple imputation resulted in an incremental effect of 0.0153 (95% CI 0.0122 to 0.0185) QALYs and a marginally reduced cost of −£4.94 (95% CI −£26.39 to 16.51), with the CRP-guided group dominating usual care with a probability of CE at WTP thresholds of £20 000 per QALY gained of 98.2%. Using the lower and upper bounds of the 95% CIs for the cost and QALY differences to conduct deterministic sensitivity analysis results in ICERs up to £10 154 with scenarios when the intervention is both dominating and dominated. This high level of uncertainty is caused by the small differences in costs and effects on utility between groups with CIs spanning zero.

## Discussion

In this comprehensive economic evaluation conducted alongside the PACE randomised controlled trial,^15 16^ we calculated a CRP-POCT cost of £11.31 per test. Use of the intervention was associated with a 20% absolute reduction in antibiotic consumption, and the POCT cost was largely offset by savings in healthcare resource use related to COPD and respiratory conditions. The mean probabilistic ICERs were £125 (95% CI −£42.00 to £518.14) per 1% absolute reduction in antibiotic consumption compared with usual care at 4 weeks and £1489 (95% CI −£61 895 to £65848) per QALY gained at 6 months, with the probability of CRP-POCT being cost-effective at a WTP threshold of £20 000 per QALY gain being 73.1%.

### Strengths and limitations

To our knowledge, this is the first UK study that has examined the CE of the use of CRP-POCT in primary care based on actual antibiotic consumption. We report on one of the few studies of antibiotic stewardship interventions that considers both a reduction in antibiotic use and no worse (non-inferior) clinical recovery.^16^

Undertaking an economic evaluation alongside a pragmatic randomised controlled trial is challenging, and we used various strategies to account for this, including handling missing data, baseline differences and skewed cost data, with extensive sensitivity analyses undertaken to account for uncertainty in our findings. Our main perspective was a UK NHS perspective, which does not consider the wider costs to the patient and society. We conducted additional analysis to take into account changes in work productivity, with no impact on our conclusions, but this is a limited presentation of a societal perspective and did not consider direct costs borne by the patient (eg, over the counter medicines) or other direct or indirect costs incurred by the patient (and family). Our CUA was sensitive to changes in HRQoL reported. This is caused by the small difference in utilities between the two groups. The PACE trial was designed to show non-inferiority in secondary outcomes, including health status and HRQoL, despite reduction in antibiotic consumption, rather than superiority. As such, small utility differences had to be expected. The short-term trial follow-up is a limitation and does not take into account the longer-term costs and outcomes associated with a chronic health condition whereby AECOPD is likely to reoccur. Alongside this, our analysis does not consider the wider health and healthcare resource implications associated with better antibiotic stewardship, and it is likely this is where potential health benefits could occur, for example, reduced antibiotic resistance hence reduced resource use in the wider population.

### Comparison with previous evidence

We identified four studies that assessed the CE of CRP-POCT for antibiotic prescribing in patients with lower respiratory tract infections, reporting similarly small cost and quality of life differences between groups.^26^,^29^ While these studies were conducted in different patient populations and settings, the similarities in the direction and magnitude of results confirm the robustness and accuracy of the CE evidence presented here. Furthermore, a model-based CUA based on data reported in the literature^30^ corroborates our findings that point-of-care testing is cost-effective in guiding antibiotic prescribing decisions for patients with AECOPD.

### Implications for practice and research

Our findings, alongside the PACE clinical trial,^16^ provide clear evidence that CRP-POCT is a cost-effective option to reduce antibiotic prescribing in primary care for patients with AECOPD without affecting health outcomes or healthcare costs. The NICE has recommended the use of CRP testing to predict pneumonia in primary care.^31^ Our findings indicate that CRP-POCT is also a cost-effective intervention for management of AECOPD in primary care. Further research should address the longer-term CE of CRP-POCT in the management of AECOPD in clinical practice and the wider impact on improving outcomes as a consequence of enhanced antibiotic stewardship and reduction in antimicrobial resistance. Moreover, the CE of CRP-POCT in other healthcare settings will need to be investigated in due course to estimate its benefit beyond the UK and especially in privately funded systems and low-resource settings.

## supplementary material

10.1136/bmjopen-2024-084144 online supplemental file 1

## Data Availability

Data are available upon reasonable request.
