# Supplementary material for: Cost-effectiveness of C-reactive protein point of care testing for safely reducing antibiotic consumption for acute exacerbations of chronic obstructive pulmonary disease as part of the multicentre, parallel-arm, open, individually randomised, controlled PACE trial
Source: BMJ Open. 2024 Nov 27;14(11):e084144. doi: 10.1136/bmjopen-2024-084144 (PMC11660330; doi:10.1136/bmjopen-2024-084144)
Supplement: online supplemental file 1 [file bmjopen-14-11-s001.docx]

# **Supplementary materials**

# **TABLE A Cost components and total testing cost of CRP POCT in primary care**

| **Cost component** | **List price (£)** | **Resource use** | **Cost/test (£)** | **Notes** |
| --- | --- | --- | --- | --- |
| **Material costs** | | | | |
| Alere Afinion CRP test cartridge* | 58·00 | 1 | 3·87 | No discounts given, 15 cartridges per pack |
| Alcohol wipe^†^ | 2·00 | 1 | 0·02 | 100 wipes per pack |
| Prick needle^§^ | 7·50 | 1 | 0·08 | 100 lancets per pack |
| Gauze pads/swabs^¶^ | 15·30 | 1 | 0·06 | 250 pads per pack |
| Gloves** | 9·19 | 2 | 0·18 | 100 per pack |
| Quality control testing kits* | 36·00 | 0·13 | 1·20 | 4 per pack; 1 high level, one low level control required per 15 tests |
| Repeat samples due to invalid results^††^ | 58·00 | 0·03 | 0·13 | 10/333 tests at baseline gave error = 3·0% |
| **Capital costs** | | | | |
| Alere Afinion AS100 analyser* | 1200 | 0·0001 | 0·13 | 7 year life span (5 samples a day assumed, 260 working days per year) = 9100 samples |
| Maintenance contract* |  | none | 0·00 | No maintenance required |
| **Set-up and training** | | | | |
| Initial set-up, calibration and configuration* |  |  | 0·00 | Done by supplier before training |
| Travel and trainer cost |  |  | 0·00 | Done at GP surgery, included in purchase price |
| GP opportunity cost^§§^ | 134/hour | 45 mins | 100·50 | GMS activity including qualifications excluding direct care staff |
| Nurse opportunity cost^§§^ | 43·00/hour | 45 mins | 32·25 | including qualifications |
| **Testing** | | | | |
| Analyser switch-on and self-test*^,§§^ | 43·00/hour | 1 min | 0·14 | nurse assumed, 1 minute needed, 5 tests a day assumed |
| Sample processing (including taking sample, labelling, preparation and test start) - GP^§§^ | 199/hour | 1·15 mins | 3·81 | patient contact excluding direct care staff, 1 minute and 9 seconds average processing time*, assumed 70% of cases |
| Sample processing (including taking sample, labelling, preparation and test start) - Nurse | 43·00/hour | 1·15 mins | 0·82 | including qualifications, 1 minute and 9 seconds average processing time*, assumed 30% of cases |
| Results check and reporting (GP) | 199/hour | 0·7 mins | 2·32 |  |
| Dealing with testing errors and retesting | 199/hour | 0·03 | 0·16 | 10/333 tests at baseline required re-testing |
| **Total test cost (£)** |  |  | **11.31** |  |
| * Source: Alere direct communication  ^†^ Source: <https://www.medisupplies.co.uk/Syringes-Needles/Pre-Injection-Wipes/Cutisoft-Pre-Injection-Wipes>  ^§^ Source: <https://www.amazon.co.uk/LANCETS-00-4MM-FINGER-PRICKING-DEVICES/dp/B004PU5AO2>  ^¶^ Source: <https://www.medisupplies.co.uk/First-Aid-Safety/Gauze-Swabs/Multisorb-Gauze-Swabs>  ** Source: <https://www.scientificlabs.co.uk/product/SAF5654>  ^††^ Source: PACE trial^1,2^  ^§§^ PSSRU 2016^3^ | | | | |

1 [Bates J](https://www.ncbi.nlm.nih.gov/pubmed/?term=Bates%20J%5BAuthor%5D&cauthor=true&cauthor_uid=28969667), [Francis NA](https://www.ncbi.nlm.nih.gov/pubmed/?term=Francis%20NA%5BAuthor%5D&cauthor=true&cauthor_uid=28969667), [White P](https://www.ncbi.nlm.nih.gov/pubmed/?term=White%20P%5BAuthor%5D&cauthor=true&cauthor_uid=28969667), et al. General practitioner use of a C-reactive protein point-of-care test to help target antibiotic prescribing in patients with acute exacerbations of chronic obstructive pulmonary disease (the PACE study): study protocol for a randomised controlled trial. [*Trials*](https://www.ncbi.nlm.nih.gov/pubmed/28969667) 2017; **18(1)**: 442. doi: 10.1186/s13063-017-2144-8.

2 [Butler CC](https://www.ncbi.nlm.nih.gov/pubmed/?term=Butler%20CC%5BAuthor%5D&cauthor=true&cauthor_uid=31291514), [Gillespie D](https://www.ncbi.nlm.nih.gov/pubmed/?term=Gillespie%20D%5BAuthor%5D&cauthor=true&cauthor_uid=31291514), [White P](https://www.ncbi.nlm.nih.gov/pubmed/?term=White%20P%5BAuthor%5D&cauthor=true&cauthor_uid=31291514). C-Reactive Protein Testing to Guide Antibiotic Prescribing for COPD Exacerbations. [*N Engl J Med*](https://www.ncbi.nlm.nih.gov/pubmed/31291514) 2019; **381(2)**: 111–20.

3 Curtis L, Burns A. Unit Costs of Health and Social Care 2016. Personal Social Services Research Unit, University of Kent, Canterbury, 2016.
