# Supplementary material for: Cost-effectiveness of C-reactive protein point of care testing for safely reducing antibiotic consumption for acute exacerbations of chronic obstructive pulmonary disease as part of the multicentre, parallel-arm, open, individually randomised, controlled PACE trial
Source: BMJ Open. 2024 Nov 27;14(11):e084144. doi: 10.1136/bmjopen-2024-084144 (PMC11660330; doi:10.1136/bmjopen-2024-084144)
Supplement: Supplementary file 2 [file bmjopen-2024-084144.reviewer_comments.pdf]

PEER REVIEW HISTORY

BMJ Open publishes all reviews undertaken for accepted manuscripts. Reviewers are asked to complete a checklist review form and are provided with free text boxes to elaborate on their assessment. These free text comments are reproduced below.

ARTICLE DETAILS

Title (Provisional)

Cost-effectiveness of C-reactive protein point of care testing for safely reducing antibiotic consumption for acute exacerbations of chronic obstructive pulmonary disease as part of the multi-centre, parallel-arm, open, individually randomised, controlled PACE trial.

Authors

Sewell, Bernadette; Francis, Nick; Harris, Shaun; Gillespie, David; Bates, Janine; White, Patrick; Alam, M Fasihul; Hood, Kerenza; Butler, Christopher C.; Fitzsimmons, Deborah

---

VERSION 1 - REVIEW

**Reviewer**	**1**
**Name**	**Pakhale, Smita**
**Affiliation**	**Ottawa Hospital Research Institute, Respirology**
**Date**	**14-Feb-2024**
**COI**	**None**

This is a well conducted economic analysis within a pragmatic RCT, a challenging task indeed.

In the limitations section, you did mention about other healthcare systems than the one in UK, however, a wider, global perspective would be appreciated. There are countries with systems that are fully Universal Health Care (UHC) (E.g. Scandinavian countries), some have partial UHC (E.g. Canada where Pharma Care is not covered under UHC), and many with two-tier systems like the USA and India where part of the system is publicly funded, however, a large part is completely private where 100% expenses are out-of-pocket. Moreover, antibiotics are misused and over-used in many countries, being available over the counter. If you could shed light on these different settings in the limitations section, would be very useful, especially to patients, physicians and policy makers in low-resource settings.

**Reviewer**	**2**
**Name**	**Chong, Huey Yi**
**Affiliation**	**Cardiff and Vale University Health Board**

Date **18-Jun-2024**

COI **None**

In this paper, the cost-effectiveness of a C-reactive protein point-of-care-test (CRP-POCT) alongside usual care vs usual care alone to guide antibiotic prescribing for AECOPD was assessed. Results showed that CRP-POCT would be a cost-effective option. A few comments and clarification points

Concerning the analyses, I have a major concern on the missingness and the approach undertaken by the authors:

1. The level of missingness in the study was not reported, and it was unclear if a complete case analysis was used as the base case analysis. Please report the % missingness and justify the appropriateness of the analysis used in base case analysis.

2. The authors carried out an independent t-test to estimate the cost differences between arm. Generally, a generalised linear model adjusted for minimisation factors is used for both costs and QALYs. I was wondering why t-test was used, and not a GLM.

A few minor comments:

1. Please add information on ethics approval of the RCT.

2. I noted that costs were expressed in 2015/16 pound sterling. It may be worth updating to the latest cost year.

3. It would be more informative to include intervention costs and the total costs in Table 1.

4. I suggest presenting the main results in a table for better readability.

VERSION 1 - AUTHOR RESPONSE

Reviewer 1

- This is a well conducted economic analysis within a pragmatic RCT, a challenging task indeed.
  - *Response: Thank you so much for this comment. It is much appreciated.*

- In the limitations section, you did mention about other healthcare systems than the one in UK, however, a wider, global perspective would be appreciated. There are countries with systems that are fully Universal Health Care (UHC) (E.g. Scandinavian countries), some have partial UHC (E.g. Canada where Pharma Care is not covered under UHC), and many with two-tier systems like the USA and India where part of the system is publicly funded, however, a large part is completely private where 100% expenses are out-of-pocket. Moreover, antibiotics are misused and over-used in many countries, being available over the counter. If you could shed light on these different settings in the limitations section, would be very useful, especially to patients, physicians and policy makers in low-resource settings.

- *Response: Unfortunately, a global perspective was beyond the scope of this trial. It would indeed be interesting to comment on the implications of the intervention in different healthcare settings and healthcare financing systems. However, the trial results prove cost-effectiveness in the UK healthcare system, and while this was a relatively large multi-centre trial, it would be difficult to predict how cost-effectiveness may change in different settings (e.g. in UHC settings or OOP settings). While the results appear robust and generalisable for the UK, we would be hesitant to make global assumptions based on our results to apply them to e.g. low-resource settings, despite the obvious importance of the question. We have added a paragraph to the implications section to explain this further.*

Reviewer 2

- Concerning the analyses, I have a major concern on the missingness and the approach undertaken by the authors:
  1. The level of missingness in the study was not reported, and it was unclear if a complete case analysis was used as the base case analysis. Please report the % missingness and justify the appropriateness of the analysis used in base case analysis.
    - *Response: Our apologies for the confusion. Differences in healthcare costs between trial arms were reported based on all available cases to provide the most complete picture available. However, the base case analysis was based on adjusted differences following multiple imputation for both costs and outcomes using appropriate factors. We have made this clearer in the text now.*

- The authors carried out an independent t-test to estimate the cost differences between arms. Generally, a generalised linear model adjusted for minimisation factors is used for both costs and QALYs. I was wondering why t-test was used, and not a GLM.
    - *Response: This is a valid point. T-tests were chosen to provide the mean differences between groups as to best illustrate actual between-group differences based on available cases. While it may be argued that the sample is large enough to assume near-normality, we agree that parametric test may not be ideal to calculate CIs and p-values. Therefore, 95% confidence intervals were calculated using non-parametric bootstrapping. We have now amended the manuscript to update the p-values being derived from non-parametric Mann-Whitney U tests rather than t-tests to address the skewness of cost data.*

- Please add information on ethics approval of the RCT.
    - *Response: We have made the ethics statement more prominently in the document by adding it to the methods section.*

- I noted that costs were expressed in 2015/16 pound sterling. It may be worth updating to the latest cost year.
    - *Response: The costs were expressed in 2015/16 to accurately reflect the year of data collection. Considering the changes in practice (especially in regards to Covid-19 and more common use of digital healthcare) since the study was conducted, updating all costs to 2023/24 prices may introduce bias. We have therefore chosen to report the original cost year.*

- It would be more informative to include intervention costs and the total costs in Table 1.
    - *Response: We added the total cost to Table 1 but decided to leave the implementation cost in the text to avoid too much duplication between tables and text.*

- I suggest presenting the main results in a table for better readability.
    - *Response: We appreciate the reviewer's comment regarding readability but have chosen not to provide a table with the main results as we believe readability to be negatively affected by too much repetition between text and tables.*

VERSION 2 - REVIEW

**Reviewer**	**1**
**Name**	**Pakhale, Smita**
**Affiliation**	**Ottawa Hospital Research Institute, Respirology**
**Date**	**15-Oct-2024**
**COI**	

Thank you for addressing all comments!

**Reviewer**	**2**
**Name**	**Chong, Huey Yi**
**Affiliation**	**Cardiff and Vale University Health Board**
**Date**	**28-Oct-2024**
**COI**	

Many thanks for addressing the comments. Happy with the revision.
